# Potential and Realized Impact of Astroglia Ca^2 +^ Dynamics on Circuit Function and Behavior

**DOI:** 10.3389/fncel.2021.682888

**Published:** 2021-06-07

**Authors:** Eunice Y. Lim, Liang Ye, Martin Paukert

**Affiliations:** ^1^Department of Cellular and Integrative Physiology, University of Texas Health Science Center at San Antonio, San Antonio, TX, United States; ^2^Center for Biomedical Neuroscience, University of Texas Health Science Center at San Antonio, San Antonio, TX, United States

**Keywords:** astroglia, behavioral state, calcium, astrocyte, neuromodulation, norepinephrine, Bergmann glia, vigilance

## Abstract

Astroglia display a wide range of spontaneous and behavioral state-dependent Ca^2+^ dynamics. During heightened vigilance, noradrenergic signaling leads to quasi-synchronous Ca^2+^ elevations encompassing soma and processes across the brain-wide astroglia network. Distinct from this vigilance-associated global Ca^2+^ rise are apparently spontaneous fluctuations within spatially restricted microdomains. Over the years, several strategies have been pursued to shed light on the physiological impact of these signals including deletion of endogenous ion channels or receptors and reduction of intracellular Ca^2+^ through buffering, extrusion or inhibition of release. Some experiments that revealed the most compelling behavioral alterations employed chemogenetic and optogenetic manipulations to modify astroglia Ca^2+^ signaling. However, there is considerable contrast between these findings and the comparatively modest effects of inhibiting endogenous sources of Ca^2+^. In this review, we describe the underlying mechanisms of various forms of astroglia Ca^2+^ signaling as well as the functional consequences of their inhibition. We then discuss how the effects of exogenous astroglia Ca^2+^ modification combined with our knowledge of physiological mechanisms of astroglia Ca^2+^ activation could guide further refinement of behavioral paradigms that will help elucidate the natural Ca^2+^-dependent function of astroglia.

## Introduction

Astroglia are ubiquitous in the brain and exhibit a range of morphological structure adapted for each brain region. Star-shaped or protoplasmic astrocytes in gray matter possess minimal internal structure and are shaped by the surrounding neuropil, whereas fibrous astrocytes in white matter contain fibrils that assist integration within axon bundles (Köhler et al., 2019). Specialized astrocytes like retinal Müller cells and cerebellar Bergmann glia have distinct orientation that aligns with the stratification of nearby sensory cells and neurons, respectively (Siegel et al., 1991; Franze et al., 2007). Across all astroglia, their space-filling arrangement provides contact with a myriad of neural cell types including the vasculature (McCaslin et al., 2011). Astroglia perform dual functions of both isolating and modulating individual synaptic contacts, conceptualized in the tripartite synapse model (Araque et al., 1999), and also maintaining extensive intercellular communication through gap junctions producing an interconnected composition within the brain (Giaume and McCarthy, 1996; Theis and Giaume, 2012). Expression of numerous neurotransmitter receptors, ion channels, and transporters (Verkhratsky and Nedergaard, 2018) demonstrates that both anatomically and functionally, astroglia are primed for a role in sensing and dynamically modulating brain-wide neural networks. Astroglia do not fire action potentials and rather display a large and selective permeability to potassium, conferring the exceptionally negative resting membrane potential of −85 to −90 mV (Kuffler, 1967; Ransom and Goldring, 1973). High potassium conductance underlies their ability to buffer extracellular ions and allows modulation of neuronal activity (Wang et al., 2015; Weiss et al., 2019).

Aside from a relatively simple electrical constitution, astroglia abundantly express receptors and molecular components that produce a range of dynamic fluctuations in intracellular Ca^2+^. A list of many but not all membrane proteins enabling rise in astroglia intracellular Ca^2+^ is shown in Table 1. The following organizing principles were used: dependence on inositol-triphosphate receptor type 2 (IP_3_R2 protein or *ITPR2* gene) – whether demonstrated or expected; *in vivo* characterization – a crucial factor given that even in the most conservative *ex vivo* preparation that is acute brain slice preparation, alterations in astroglia protein expression, morphology and Ca^2+^ dynamics can occur within less than 2 h of preparation (Takano et al., 2014); use of unanesthetized animal model – since general anesthesia strongly suppresses global astroglia Ca^2+^ elevations (Nimmerjahn et al., 2009; Thrane et al., 2012); and trigger of Ca^2+^ signal – to describe whether or not astroglia signaling pathways contributing to Ca^2+^ dynamics have been studied within their physiological dynamic range. Since in most cases it is very difficult if even possible to quantify the physiological dynamic range, for the tables we take a conservative approach and consider as “physiological” only astroglia Ca^2+^ dynamics that occur spontaneously or are driven by sensory stimulation or behavioral state, which we distinguish from electrical stimulation of an axonal fiber tract, application of a receptor agonist, or activation of an exogenously expressed receptor or channel. Various exogenous proteins for manipulation of astroglia intracellular Ca^2+^ are further explored in Table 2. We follow these concepts in order to emphasize the importance of using *in vivo* experimental paradigms that preserve or at least mimic the natural condition in order to understand which properties among the many purported abilities of astrocytes are physiologically employed. For each experiment, if concepts two or three are met, they are represented in green, and if not, they are represented in orange or without color. The more concepts are represented in green, we consider the respective finding to demonstrate a realized astroglia function. Conversely, the fewer concepts are represented in green, we consider that the respective finding may indicate a potential astroglia function.

**TABLE 1:** Membrane proteins controlling *in situ* astroglia intracellular Ca^2+^.

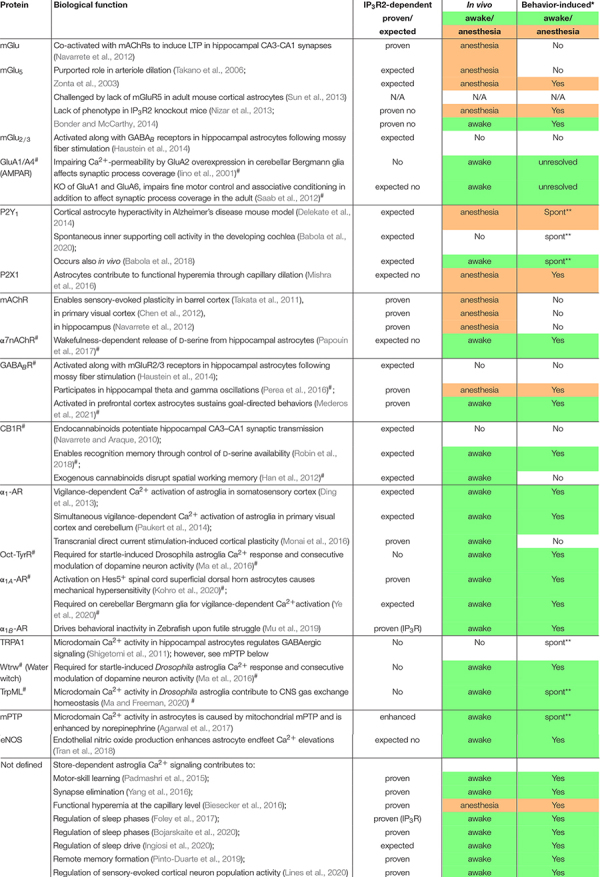

Proteins are described in terms of the following: biological function; proven or expected dependence on IP_3_R2; whether experiments were conducted in vivo and if so, whether done in awake (green) or anesthetized (orange) mice; and whether the Ca^2+^ signal is behavior-induced and if so, whether this was validated in awake (green) or anesthetized (orange) mice. ^#^, indicates proteins for which functional expression in astroglia has been demonstrated by conditional gene deletion or rescue, and the respective studies are indicated individually. *, “Behavior-induced” indicates that a Ca^2+^ signal is caused by any form of sensory stimulation or awake behavior. Accepted manipulations are forms of acute or chronic suppression of endogenous signaling pathways. Any form of electrical, optical, or chemical overactivation of a nerve fiber tract or an endogenous or exogenous signaling pathway does not qualify. **, “spont” or spontaneous indicates Ca^2+^ signal is unprovoked and not triggered by a specific behavioral state.

*mGlu, metabotropic glutamate receptor; GluA, AMPA-type glutamate receptor; P2Y_1_, P2Y purinoceptor 1; P2X1, P2X purinoceptor 1; mAChR, muscarinic acetylcholine receptor; α7nAChR, α7 nicotinic acetylcholine receptor; GABA_*B*_R, metabotropic GABA_*B*_ receptor; CB1R, cannabinoid receptor 1; α_1_-AR, α_1_-adrenergic receptor; Oct/TyrR, tyramine/octopamine receptor; TRPA1, transient receptor potential A1 channel; Wtrw, transient receptor potential channel Water witch; TrpML, transient receptor potential mucolipin channel (vertebrate orthologs TRPML1-3 or mucolipins 1-3); mPTP, mitochondrial permeability transition pore; eNOS, endothelial nitric oxide synthase*.

**TABLE 2:** Exogenous proteins for manipulation of astroglia intracellular Ca^2+^.

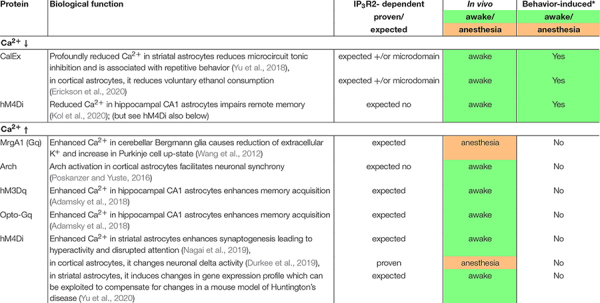

Exogenous manipulations are described using the same principles as in Table 1: biological function; proven or expected dependence on IP_3_R2; whether experiments were conducted in vivo and if so, whether done in awake (green) or anesthetized (orange) mice; and whether the Ca^2+^ signal is behavior-induced and if so, whether this was validated in awake (green) or anesthetized (orange) mice. * “Behavior-induced” indicates that a Ca^2+^ signal is caused by any form of sensory stimulation or awake behavior. Accepted manipulations are forms of acute or chronic suppression of endogenous signaling pathways. Any form of electrical, optical, or chemical overactivation of a nerve fiber tract or an endogenous or exogenous signaling pathway does not qualify.

We intentionally do not categorize astroglia Ca^2+^ dynamics by somatic or process location since, as we will discuss, a considerable portion of process Ca^2+^ elevations share the underlying mechanism with somatic Ca^2+^ elevations. Also, it is noted that astroglia Ca^2+^ signaling has been tightly associated with the concept of gliotransmission, which is the Ca^2+^-dependent vesicular release of signaling molecules from astroglia (Araque et al., 2014). The existence and/or significance of this concept still faces controversy (Fiacco and McCarthy, 2018; Savtchouk and Volterra, 2018). It is beyond the reach of this review to resolve this controversy. Instead, we take an agnostic glial neuromodulation view at what has been found about astroglia Ca^2+^ dynamics in living animals and how it is or may be linked to neuronal activity and behavior.

In this review, we describe the various forms of astroglia Ca^2+^ signaling distinguishing between (i) whole-cell, behavior-associated signals as well as (ii) spatially localized microdomain activity, and (iii) the functional consequences of their inhibition. We then discuss (iv) what effects of exogenous astroglia Ca^2+^ activation have been identified that could guide further refinement of behavioral paradigms to study the natural function of astroglia.

## Mechanisms of Global, Behavior-Associated Astroglia Ca^2+^ Activation

Relating mammalian astroglia Ca^2+^ dynamics directly to behavioral state became possible with the introduction of an approach that allows study of cellular brain activity in head-fixed awake mice on a spherical treadmill using two-photon microscopy (Dombeck et al., 2007). It was found that protoplasmic astrocytes in the somatosensory cortex produce Ca^2+^ transients that correlate with bouts of motor activity. The head-fixation two-photon microscopy paradigm was soon adopted to study cerebellar Bergmann glia (Nimmerjahn et al., 2009). Three distinct types of Ca^2+^ signals were defined in this seminal study. First, ‘bursts’ are spontaneous radially spreading Ca^2+^ elevations that depend on purinergic signaling and can also be detected in anesthetized rats and mice (Hoogland et al., 2009). Since they are not at all, or extremely rarely detected in awake mice through chronic cranial windows after sufficient tissue recovery from surgery (Paukert et al., 2014), they may be triggered by trauma-associated ATP release. Second, ‘sparkles’ are spontaneous, low-frequency Ca^2+^ elevations that occur asynchronously in individual Bergmann glia process domains and persist at lower frequency under isoflurane anesthesia. These events may correspond to microdomain Ca^2+^ activity in protoplasmic astrocytes which will be discussed below. Finally, ‘flares’ are a robust, whole-cell Ca^2+^ transient encompassing Bergmann glia in the entire field of view, are detectable only in awake mice, and relate to the onset of motor activity.

The underlying mechanism of this motor activity-associated astroglia Ca^2+^ elevation remained elusive in these initial studies. It is now known that locomotion is associated with sufficient arousal to trigger the release of norepinephrine and acetylcholine. In mouse primary visual cortex for example, noradrenergic signaling is responsible for tonic depolarization of the membrane potential of layer 2/3 and 4 excitatory neurons as well as interneurons during locomotion (Polack et al., 2013). Locomotion also evokes a Ca^2+^ rise in vasoactive intestinal peptide (VIP)-positive neurons, which depends on activation of nicotinic acetylcholine receptors (Fu et al., 2014). Finally, direct observation of terminal Ca^2+^ dynamics was accomplished by expression of the genetically-encoded Ca^2+^ indicator (GECI) GCaMP6s in mouse noradrenergic and cholinergic neurons, respectively. Locomotion is accompanied by Ca^2+^ elevations in both types of terminals in primary visual and auditory cortices (Reimer et al., 2016).

Electrical stimulation of brainstem nuclei in anesthetized mice revealed the powerful potential of neuromodulators for coordinated Ca^2+^ activation of astroglia. Stimulation of locus coeruleus triggers norepinephrine release and leads to subsequent Ca^2+^ activation of somatosensory cortex astrocytes (Bekar et al., 2008). Stimulation of nucleus basalis of Meynert induces acetylcholine release and causes a Ca^2+^ activation of astrocytes in somatosensory cortex (Takata et al., 2011), primary visual cortex (Chen et al., 2012), and hippocampus (Navarrete et al., 2012). Consistent with the idea that vigilance-dependent astroglia Ca^2+^ activation may be mediated by norepinephrine, it was found that locomotion as well as isometric muscle contractions, such as during a freezing response, induce widespread Ca^2+^ elevations in mouse cerebellar Bergmann glia, an occurrence previously described as ‘flares.’ Vigilance-dependent Bergmann glia responses are highly correlated with astrocyte Ca^2+^ elevations in primary visual cortex (Paukert et al., 2014). In the same study, pharmacological experiments revealed the importance of α_1_-adrenergic receptors for Bergmann glia Ca^2+^ activation. α_1_-adrenergic receptors had been independently found to be required for whisker stimulation- and air puff-induced Ca^2+^ activation of mouse somatosensory cortex astrocytes (Ding et al., 2013). Notably, locomotion-induced widespread Bergmann glia Ca^2+^ elevations have been studied in the molecular layer of cerebellar cortex where cross-sections of Bergmann glia processes are captured (Nimmerjahn et al., 2009; Paukert et al., 2014). Consistent with this finding, locomotion/startle-induced Ca^2+^ elevations in cortical astrocytes encompass processes in addition to somata (Paukert et al., 2014; Srinivasan et al., 2015; Ye et al., 2017; King et al., 2020).

Recently, use of global as well as cell type-specific gene deletion revealed that α_1*A*_-adrenergic receptors on Bergmann glia are required for vigilance-dependent Ca^2+^ activation (Ye et al., 2020). The generated conditional knockout mouse line in combination with the inducible *Aldh*1*l*1−*CreER*^*T*2^ mouse line (Srinivasan et al., 2016) led to a loss of >94% of *Adra1a* mRNA in the cerebellum, indicating that within the cerebellum, α_1*A*_-adrenergic receptors are potentially exclusively expressed in astroglia. Despite almost complete loss of Bergmann glia Ca^2+^ elevations in the global *Adra1a* knockout mouse, the loss of responsiveness in the conditional mouse more than 1 month following Cre recombination was approximately 40%, suggesting an extended stability of some receptors and highlights an obstacle for the interpretation of negative behavioral data. An independent study found that knock-out of α_1*A*_-adrenergic receptors in mouse *Hes5*-positive astrocytes of superficial laminae of the spinal dorsal horn strongly reduced Ca^2+^ elevations in those cells in response to intraplantar injection of capsaicin (Kohro et al., 2020).

Noradrenergic signaling to astroglia is evolutionarily highly conserved. Indeed, the first experimental evidence for direct signaling of neuromodulators through astroglia was obtained from studying *Drosophila* astrocytes (Ma et al., 2016). Olfactory-driven larval chemotaxis as well as touch-induced startle responses induce somatic Ca^2+^ elevations in astrocytes that are mediated by the release of the *Drosophila* norepinephrine analogs tyramine and octopamine from Tdc2-positive neurons through binding to their receptor on astrocytes. In zebrafish, radial astrocytes accumulate information about futile motor activity through α_1*B*_-adrenergic receptors (Mu et al., 2019). Astroglia Ca^2+^ elevations encompassing whole cells can also occur spontaneously. In the developing mouse cochlea, clusters of supporting cells undergo Ca^2+^ elevations that depend on the activation of P2Y_1_ receptors (Tritsch et al., 2007; Babola et al., 2020). Astroglia express many more G protein-coupled receptors (Kofuji and Araque, 2020). Most if not all of them contribute to the diverse pool of astroglia microdomain Ca^2+^ dynamics that will be discussed in the next section. It is currently unclear and awaits further investigation whether they also modulate norepinephrine-mediated, vigilance-dependent global astroglia Ca^2+^ elevations, independently cause global astroglia Ca^2+^ elevations in yet to be defined behavioral context, or exclusively contribute to microdomain activity.

## Mechanisms Leading to Microdomain Ca^2+^ Dynamics in Astroglia

The astroglia network is anatomically complex and dynamic. A single astroglia contacts multiple neuronal cell bodies, hundreds of processes, and tens of thousands of synapses (Ventura and Harris, 1999). In addition, a considerable body of experimental findings suggests functional interactions between astrocytes and neurons at the synaptic level, termed gliotransmission, most of which have been determined to depend on astrocyte Ca^2+^ elevations (Araque et al., 2014). This feature has inspired investigations in subcellular, localized Ca^2+^ dynamics restricted to small portions of astroglia processes. First observed in acute cerebellar slices, ‘microdomain’ Ca^2+^ transients in Bergmann glia processes occur spontaneously or in response to electrical stimulation of parallel fibers, which are glutamatergic axons of granule cells passing by Purkinje cell dendrites, molecular layer interneurons and Bergmann glia processes (Grosche et al., 1999). Bergmann glia express GluA2 lacking, Ca^2+^-permeable α-amino-3-hydroxy-5-methyl-4-isoxazolepropionic acid (AMPA) type glutamate receptors (Burnashev et al., 1992; Müller et al., 1992) and the G_*q*_ protein-coupled purinergic receptor P2Y_1_ (Rudolph et al., 2016). The fast component of Ca^2+^ elevation following parallel fiber stimulation has been ascribed to AMPA receptors (Piet and Jahr, 2007) and a second slower component of Ca^2+^ elevation depends on mGlu_1_-dependent release of ATP from molecular layer interneurons (Beierlein and Regehr, 2006; Piet and Jahr, 2007).

In awake mice, the Bergmann glia Ca^2+^ signal equivalent of microdomain events was previously termed ‘sparkles’ (Nimmerjahn et al., 2009). They are spontaneous Ca^2+^ elevations spatially restricted to individual main process branches that anatomically appear as palisades. Sparkles persist at a lower frequency during inhibition of action potential firing with tetrodotoxin (TTX), during inhibition of glutamatergic receptors, and during isoflurane general anesthesia. These features suggest that a considerable portion of sparkles resemble intrinsically-generated Ca^2+^ elevations that have been previously described in cortical astrocytes of anesthetized mice (Takata and Hirase, 2008).

Two major experimental obstacles have limited the appreciation of microdomain Ca^2+^ dynamics. The paucity in cytosolic volume of the finest astroglia processes limits the availability of synthetic Ca^2+^ indicators as well as cytosolic GECIs in those compartments and reduces the signal-to-noise level. The other difficulty is in distinguishing between a microdomain Ca^2+^ event and global Ca^2+^ signaling events spreading into the microdomain’s spatial territory. For instance, locomotion-associated ‘flares’ prevent determining if ‘sparkles’ are enhanced during locomotion (Nimmerjahn et al., 2009). A significant advancement came with the introduction of membrane-tethered GECIs (Shigetomi et al., 2010a,b), which detect miniature Ca^2+^ fluctuations in astroglia localized near the plasma membrane and within the finest processes with only minimum volume of cytoplasm. These ‘spotty’ Ca^2+^ transients have more variable kinetics including faster responses than global vigilance-dependent responses described above, and occur asynchronously (Srinivasan et al., 2015; Agarwal et al., 2017). Some asynchrony of microdomain Ca^2+^ events may be explained by the biochemical compartmentation of nodal structural elements often localized in proximity to dendritic spines, a discovery recently made with super-resolution microscopy within the meshwork of astrocyte fine processes (Arizono et al., 2020).

By expressing the membrane-anchored GECI in acute hippocampal slices and organotypic culture, respectively, it was deduced that TRPA1 is required for astrocyte microdomain responses and for setting the basal Ca^2+^ level (Shigetomi et al., 2011, 2013; Jackson and Robinson, 2015). Using transgenic mice expressing a membrane-anchored GECI in astrocytes confirmed that TRPA1 contributes to the basal hippocampal astrocyte Ca^2+^ level, but transient microdomain Ca^2+^ events are not sensitive to pharmacological inhibition of TRPA1, Ca^2+^ release-activated channels (CRACs), ryanodine receptors, voltage-dependent Ca^2+^ channels or Na^+^-Ca^2+^ exchangers (Agarwal et al., 2017). Instead, consistent with the considerable subcellular co-localization of microdomain Ca^2+^ events and mitochondria (Jackson and Robinson, 2015; Agarwal et al., 2017), inhibition of the mitochondrial permeability transition pore (mPTP) dramatically reduces the frequency of microdomain Ca^2+^ events (Agarwal et al., 2017). Stochastic opening of the mPTP causes brief bursts of reactive oxygen species (ROS) production in mitochondria (Wang et al., 2008). In turn, excessive ROS production can open mPTP (Vercesi et al., 1997; Duchen, 2000). Remarkably, microdomain Ca^2+^ events, like global, vigilance-dependent Ca^2+^ responses, are also evolutionarily conserved. In *Drosophila* astrocytes, ROS-regulated TrpML is responsible for transient microdomain Ca^2+^ elevations (Ma and Freeman, 2020). ROS facilitate IP_3_R-mediated Ca^2+^ release from stores (Bánsághi et al., 2014), and further promote mPTP opening (Agarwal et al., 2017).

IP_3_R2 plays an important role for microdomain Ca^2+^ events because the frequency of those events is strongly reduced in *ITPR2^–/–^* mice (Srinivasan et al., 2015; Agarwal et al., 2017). In the absence of any circuit stimulation, IP_3_R-dependent facilitation of microdomain Ca^2+^ events appears to be not dependent on vesicular neurotransmitter release since incubation with veratridine and bafilomycin A1, for depleting vesicle content, does not affect the frequency of those events (Agarwal et al., 2017). On the other hand, it has been demonstrated that electrical stimulation of individual hippocampal axons in acute slices can evoke microdomain Ca^2+^ elevations (Bindocci et al., 2017) and that whisker stimulation, air puff stimulation, and locomotion can facilitate microdomain Ca^2+^ events (Srinivasan et al., 2015; Agarwal et al., 2017; Stobart et al., 2018). Certainly, it needs to be noted that, as mentioned above, it may be difficult to distinguish between highly coordinated microdomain Ca^2+^ activity and predominantly IP_3_R2-dependent global whole cell Ca^2+^ activity. Intriguingly, sensory stimulation and arousal can enhance microdomain activity even in *ITPR2^–/–^* mice, and norepinephrine can still enhance microdomain event frequency in the absence of IP_3_R2 (Agarwal et al., 2017) but the α_1_-adrenergic receptor antagonist prazosin does not inhibit this enhancement (Srinivasan et al., 2015). Thus, if this is a direct effect of norepinephrine on astrocytes through non-type 2 IP3 receptors (Sherwood et al., 2017), it is possible that α_2*A*_-adrenergic receptors, whose mRNA is expressed in astrocytes (Zhang et al., 2014), could contribute to facilitation of microdomain Ca^2+^ events.

## Consequences of Astroglia-Specific Deletion of Endogenous Plasma Membrane Proteins

The most convincing and complete evidence if and how astroglia Ca^2+^ dynamics influence circuit activity and behavior stems from experimental approaches that allow a combination of the following three components: (1) visualization of astroglia Ca^2+^ dynamics in awake organisms during behavior, (2) reduction or abolishment of the behavior-dependence of Ca^2+^ dynamics through astroglia-specific genetic deletion of a signaling pathway component, and (3) the detection of consequences for circuit activity and/or behavior.

### Noradrenergic Signaling

Employing such a three-component approach has revealed the crucial role of noradrenergic signaling in the context of widespread whole-astrocyte Ca^2+^ elevations. Its importance is further demonstrated by its evolutionary conservation. Sensory stimulation (olfaction) or startling touch of *Drosophila* larvae triggers release of the *Drosophila* adrenergic transmitter analogs octopamine and tyramine from Tdc2-positive neurons (Ma et al., 2016). Octopamine/tyramine (Oct/Tyr) bind directly to astrocyte Oct/TyrR causing extracellular Ca^2+^ influx through the transient receptor potential (TRP) channel Water witch (Wtrw). Somatic astrocyte Ca^2+^ elevation, through an unknown mechanism, potentially causes release of ATP, which following breakdown to adenosine, activates the inhibitory AdoR on dopaminergic neurons, relieving chemotaxis and startle-induced reversal behaviors from dopaminergic inhibition (Ma et al., 2016). Notably, this study provided the first experimental evidence that neuromodulators can signal directly through astroglia. In addition to *Drosophila*, futile motor activity in zebrafish causes incremental Ca^2+^ elevations in radial astrocytes ultimately leading to a ‘giving up’ change in behavioral state (Mu et al., 2019). Mismatch between swimming as motor action and visual virtual feedback, a paradigm previously employed in studies of mouse primary visual cortex (Keller et al., 2012), activates noradrenergic neurons and leads to a delayed global Ca^2+^ elevation in radial astrocytes. This astrocyte Ca^2+^ activation is dependent on α_1*B*_-adrenergic receptors and through the combination of opto-/chemogenetics and laser cell ablation it has been found that astrocyte Ca^2+^ activation, through a yet unknown mechanism, activates inhibitory neurons that trigger a cessation of swimming attempts (Mu et al., 2019).

In mouse cerebellar Bergmann glia, it is α_1*A*_-adrenergic receptors that are responsible for vigilance-dependent Ca^2+^ activation (Ye et al., 2020). Remarkably, despite almost complete loss of locomotion-induced Bergmann glia Ca^2+^ elevations in global *Adra1a* knockout mice, the mice do not display any motor coordination deficit. Consistent with this finding, acute exposure to ethanol (2 g/kg i.p.), which suppresses locomotion-induced norepinephrine release, results in considerable loss of Bergmann glia Ca^2+^ responsiveness that lasts for more than 45 min whereas ataxic motor coordination, present 15 min after onset of exposure to ethanol, is completely recovered 30 min later (Ye et al., 2020). These findings suggest that vigilance-dependent Bergmann glia global Ca^2+^ elevations may play a role in cognitive function of the cerebellum rather than motor coordination; however, it needs to be noted that direct evidence for this is still missing.

An independently developed *Adra1a* conditional knockout mouse line was used to investigate the role of α_1*A*_-adrenergic receptors in *Hes5*-positive astrocytes in superficial laminae of the spinal dorsal horn (supSDH) (Kohro et al., 2020). Although the completeness of reduction of ipsilateral, intraplantar capsaicin-induced *Hes5*-positive supSDH astrocyte Ca^2+^ elevation in *Adra1a* conditional knockout mice was not tested, strikingly, it was found that mechanosensory hypersensitivity was abolished. The precise mechanism how α_1*A*_-adrenergic receptor activation in *Hes5*-positive supSDH astrocytes triggers mechanosensory hypersensitivity remains elusive. One possible mechanism is based on the concept of gliotransmission where D-serine, besides glutamate and ATP, is the most frequently proposed signaling molecule (Yang et al., 2003; Panatier et al., 2006; Henneberger et al., 2010), but see Wolosker et al. (2016), Mothet et al. (2019). D-serine may function as a diffusible messenger following exogenous activation of P2Y_1_ receptors on lamina I SDH astrocytes to spread gliogenic long-term potentiation (LTP) beyond individual synapses (Kronschläger et al., 2016). It is conceivable that α_1*A*_-adrenergic receptors in *Hes5*-positive supSDH astrocytes could substitute the experimental role of P2Y_1_ receptors and control D-serine signaling. Indeed, Kohro et al. (2020) found that exogenously administered D-serine is sufficient to cause mechanosensory hypersensitivity. However, they did not detect extracellular elevations of D-serine in *Hes5*-positive supSDH astrocytes even following the drastic activation of a Designer Receptors Exclusively Activated by Designer Drugs (DREADD), specifically the humanized G_*q*_ protein-coupled muscarinic receptor type 3-based DREADD (hM3Dq). This finding could either mean that even excessive Ca^2+^ elevation in *Hes5*-positive supSDH astrocytes does not modulate extracellular D-serine signaling, or it could suggest that the detection method was not sensitive enough to take advantage of the *Adra1a* conditional knockout mouse line to imperatively link capsaicin-induced, noradrenergic Ca^2+^ activation of *Hes5*-positive supSDH astrocytes to D-serine signaling.

Robust noradrenergic signaling to astroglia in awake mice establishes a translational potential. Monai et al. (2016) found that a 10 min episode of transcranial direct current stimulation (tDCS), which is employed to ameliorate symptoms in patients of major depression among other neuropsychiatric and neurological conditions, when applied to mouse visual cortex, induces an α_1_-adrenergic receptor-dependent widespread, global Ca^2+^ elevation in astrocytes but not in neurons (Monai et al., 2016). Despite the absence of tDCS-induced neuronal Ca^2+^ elevation, a single 10 min tDCS application is sufficient for IP_3_R2-dependent induction of an hours-lasting potentiation of visually evoked potentials.

### Cholinergic Signaling

For many astroglia receptors it remains to be defined in which awake behavioral context they are activated. This requires investigation of whether they lead to global astroglia Ca^2+^ activation like noradrenergic receptors, whether they potentiate noradrenergic responses, or whether they exclusively contribute to microdomain Ca^2+^ events. For example, there is strong evidence that electrical stimulation of cholinergic nuclei and fibers in anesthetized mice leads to muscarinic receptor-dependent global Ca^2+^ elevation in somatosensory cortex, primary visual cortex and hippocampal astrocytes affecting local circuit plasticity (Takata et al., 2011; Chen et al., 2012; Navarrete et al., 2012). Given that cholinergic signaling is engaged during states of heightened vigilance (Polack et al., 2013; Fu et al., 2014; Reimer et al., 2016), it is somewhat surprising that no widespread, heightened vigilance-dependent global cortical astrocyte Ca^2+^ elevations persist following pharmacological inhibition of α_1_-adrenergic receptors (Ding et al., 2013; Srinivasan et al., 2015). Also, indirect evidence from a detailed study of sleep states suggests that acetylcholine may not directly lead to a Ca^2+^ rise in cortical astrocytes (Bojarskaite et al., 2020) though a fraction of somatosensory cortex astrocyte microdomain Ca^2+^ events in awake mice is sensitive to the muscarinic receptor antagonist atropine (Stobart et al., 2018). This highlights that caution is warranted when electrical stimulation of fiber tracts is employed. For instance, with cerebellar granule cell-Purkinje cell glutamatergic synapses, whether one stimulates bundles of neighboring axons in the molecular layer or whether one excites dispersed axons by stimulating in the granule cell layer determines if glutamate spillover leads to endocannabinoid-dependent short-term plasticity (Marcaggi and Attwell, 2005). For this reason, in Tables 1, 2 we emphasize the difference between behavior-induced (sensory stimulation or behavioral state) versus electrical stimulation- or excitatory opto- or chemogenetic stimulation-induced astroglia Ca^2+^ dynamics.

A very elegant study revealed that wakefulness/vigilance-dependent release of acetylcholine leads to circadian oscillations in hippocampal extracellular D-serine levels (Papouin et al., 2017). D-serine circadian oscillations were abolished when the gene for α7 nicotinic acetylcholine receptors (nAChRs) was specifically deleted from hippocampal CA1 astrocytes. Extracellular L-serine does not follow circadian oscillations, indicating that circadian D-serine fluctuations originate from astrocytes. Remarkably, α7nAChR-dependent D-serine release causes the glycine-binding site of *N*-methyl D-aspartate receptors (NMDARs) to reach saturation during the active/wakeful phase of the mouse. This finding raises the possibility that transient vigilance-independent D-serine release may be less impactful during the active phase, when NMDAR glycine-binding sites appear to be saturated, than during sleep, and may offer an explanation why several phenotypes arising from astroglia-selective reductions in Ca^2+^ dynamics have been found linked to the regulation of sleep (Halassa et al., 2009; Foley et al., 2017; Bojarskaite et al., 2020; Ingiosi et al., 2020).

### Glutamatergic Signaling

Glutamatergic signaling to astrocytes has been intensively studied in juvenile rodent hippocampus initially with a significant emphasis on metabotropic glutamate receptor mGlu_5_ signaling (Rose et al., 2018). *In vivo* topical pharmacology experiments have suggested that mGlu_5_ activation on somatosensory cortex astrocytes contributes to contralateral forepaw stimulation- or local electrical stimulation-induced arteriole dilation leading to functional hyperemia (Zonta et al., 2003; Takano et al., 2006). However, while this does not exclude mGlu_5_ signaling in astrocytic endfeet where microdomain Ca^2+^ events are spared, it has been demonstrated that in *ITPR2^–/–^* mice where the majority of Ca^2+^ elevations in soma and main processes as well as a considerable portion of fine process microdomain events are abolished, functional hyperemia is not impaired (Nizar et al., 2013; Bonder and McCarthy, 2014). More recent work has even revealed the reverse flow of information: cortical astrocyte endfeet in contact with arterioles sense functional hyperemia-associated vasodilation and endothelial nitric oxide production and respond with enhanced Ca^2+^ elevations (Tran et al., 2018).

Developmental expression analysis of rodent mGlu_5_ in mouse cortical astrocytes revealed steep downregulation during the third and fourth postnatal weeks leaving the G_*i/o*_ protein-coupled receptor mGlu_2/3_ as the predominant astrocyte metabotropic glutamate receptor (Sun et al., 2013). The developmental downregulation of cortical astrocyte mGlu_5_ expression is also subject to pathological dysregulation. In a mouse model of Fragile X syndrome, microRNA miR-128-3p is expressed at an elevated level in astroglia and causes accelerated loss of mGlu_5_ function (Men et al., 2020). Similar to cortical astrocyte downregulation of mGlu_5_ expression, adult mouse hippocampal mossy fiber stimulation-evoked astrocyte Ca^2+^ elevations as well as the frequency of spontaneous microdomain Ca^2+^ events are not inhibited by the antagonist for mGlu_5_ but are sensitive to antagonists for mGlu_2/3_ and for the metabotropic γ-amino butyric acid (GABA) receptor GABA_*B*_R (Haustein et al., 2014). In contrast to their action in neurons, G_*i/o*_ protein-coupled receptors in astroglia often enhance Ca^2+^ elevations, most likely through direct initiation and/or facilitation of IP_3_R opening by the βγ subunit (Zeng et al., 2003; Durkee et al., 2019; Nagai et al., 2019), but see (Kol et al., 2020).

Regarding *in vivo* consequences of glutamatergic signaling to astroglia, AMPA receptor function in cerebellar Bergmann glia is among the better understood examples. Two distinct strategies have been pursued to reduce or eliminate AMPA receptor-mediated Ca^2+^ influx. As demonstrated in one study, viral overexpression of the GluA2 subunit in Bergmann glia renders AMPA receptors Ca^2+^ impermeable, impairs the close ensheathment of glutamatergic synapses onto Purkinje cells by Bergmann glia processes and consequently, slows glutamate clearance (Iino et al., 2001). Developmental pruning of the climbing fiber, one of the two major excitatory inputs to the cerebellar cortex, is also impaired. The second strategy was use of conditional knockout of the two predominant AMPA receptor subunits expressed in Bergmann glia, GluA1 and GluA4. Twelve weeks after inducing gene deletion, Bergmann glia processes were morphologically altered, synaptic glutamate clearance impaired, and miniature excitatory postsynaptic currents in Purkinje cells were less frequent (Saab et al., 2012). Moreover, the mice exhibited a higher propensity toward missteps on the Erasmus Ladder, which is a fine motor coordination task, and impaired eyeblink conditioned responses. Together, these findings indicate that Ca^2+^ influx through AMPA receptors is an important signal for establishment and maintenance of proper synaptic function in the cerebellar cortex.

### Purinergic Signaling

ATP is among the most reliable agonists to trigger global Ca^2+^ elevations as well as enhance microdomain Ca^2+^ events in astroglia *in vitro*. *In vivo*, some of the best understood mechanisms of purinergic signaling through astroglia are during development, pathological reactivity and functional hyperemia. Before hearing onset, astroglia-like inner supporting cells (ISCs) spontaneously release ATP which activates P2Y_1_ autoreceptors and together with intense gap junction coupling among ISCs, intracellular Ca^2+^ rises in clusters of neighboring cells (Babola et al., 2020). The Ca^2+^ elevation leads to opening of Ca^2+^-activated chloride channel TMEM16A which extrudes chloride, drives potassium efflux, and causes transient depolarization of neighboring inner hair cells (Wang et al., 2015). Bursts of action potentials in spiral ganglion neuron (SGN) afferents that innervate neighboring inner hair cells propagate throughout the pre-hearing auditory pathway preserving later tonotopic representation (Babola et al., 2018). ISC-initiated transient potassium elevations can even excite neighboring SGN afferents directly when inner hair cell-SGN afferent synaptic transmission is impaired (Babola et al., 2018). It is thought that this astroglia-mediated spontaneous and transient activation of the auditory pathway allows for circuit maturation so that sensory processing may begin when the ear canal opens.

In adult rodents, P2Y_1_ receptors participate in astrocyte-to-neuron signaling in brainstem-mediated baroreception and response. It has been found that experimentally increasing intracranial pressure leads to increased venous pressure, reduced difference in brain arterio-venous pressure, and decreases the brain blood flow, which ultimately causes a Ca^2+^ elevation in brainstem astrocytes that are in proximity to central sympathetic autonomous nervous system centers (Marina et al., 2020). In response, the heart rate and the mean arterial pressure increase for homeostatic regulation of brain blood flow. This astrocytic regulatory response was impaired when vesicular release was hampered by overexpressing dominant-negative soluble *N*-ethyl-maleimide-sensitive factor attachment protein receptor (dn-SNARE). The Gourine laboratory studied the pressure sensing mechanism in more detail in an *in vitro* preparation and proposed a model for the initial pressure-dependent ATP release mechanism based on an interaction between TRPV4 ion channels and connexin 43. After finding that P2Y_1_ autoreceptors appear to serve as signal amplifiers, they deduced that vesicular release of ATP from brainstem astrocytes is the signal that excites central sympathetic centers (Turovsky et al., 2020). Astrocyte P2Y_1_ receptors have also been found to account for the enhanced microdomain Ca^2+^ event frequency in cortical astrocytes of anesthetized mouse models of Alzheimer’s disease (Kuchibhotla et al., 2009; Delekate et al., 2014).

Ionotropic receptors are likewise utilized by astrocytes to receive purinergic signals. Functional hyperemia occurs differently at the capillary level, which is controlled by astroglia Ca^2+^ dynamics, and the arteriole level, which occurs through interneuron nitric oxide release (Mishra et al., 2016). Astroglia-mediated, neuronally evoked dilation of capillaries occurs when Ca^2+^ influx through P2X1 receptors activates phospholipase D2 and diacylglycerol lipase to generate arachidonic acid. The regulation of functional hyperemia in the rat retina (Biesecker et al., 2016) is consistent with the concept of astroglia mediation of functional hyperemia at the level of capillaries (Mishra et al., 2016) but not arterioles (Nizar et al., 2013; Bonder and McCarthy, 2014). Light flickering induces Ca^2+^ elevations in Müller cell fine processes in contact with capillaries and in those in contact with arterioles. In *ITPR2^–/–^* mice with impaired light flickering-induced Ca^2+^ elevations in both types of fine processes, vasodilation is impaired only with capillaries (Biesecker et al., 2016).

### Cannabinergic Signaling

Circuit and behavior analysis following astroglia-specific gene deletion of the cannabinoid receptor type 1 (CB1R) settled an earlier controversy about the expression of this receptor in astrocytes (Stella, 2004; Han et al., 2012). In addition to mGlu_2/3_ receptors and GABA_*B*_ receptors, G_*i/o*_ protein-coupled CB1R activation also leads to astrocyte Ca^2+^ elevations (Navarrete and Araque, 2008, 2010; Robin et al., 2018). It still remains to be determined whether in awake behaving mice CB1R activation leads to global, whole-cell Ca^2+^ elevation as seen in slice experiments following neuronal depolarization or CB1R agonist application, or to increased frequency of microdomain Ca^2+^ events.

Depending on intensity and duration of hippocampal astrocyte CB1R activation, different effects on local circuit activity and behavior have been observed. Han et al. (2012) tested the hypothesis that astrocyte CB1Rs could account for the known detrimental effect of acute cannabinoid exposure for spatial working memory (Lichtman and Martin, 1996). They found that acute cannabinoid exposure is required during induction, but not for expression of *in vivo* hippocampal CA3-CA1 long-term depression (LTD) (Han et al., 2012). Cannabinoids cause LTD through astrocyte CB1R but not through glutamatergic or GABAergic neuron CB1R. LTD occurs through internalization of postsynaptic AMPA receptors that is independent of mGlu1 activation but dependent on NMDA receptor activation. The impairment in spatial working memory follows the same pharmacological profile as the impairment of astrocyte CB1R-mediated LTD (Han et al., 2012). A recent study on NMDA receptor-dependent, postsynaptic expression of CA3-CA1 hippocampal LTD further demonstrated that this circuit phenomenon depends on the astrocytic activation of the p38α variant of mitogen-activated protein kinase (MAPK) (Navarrete et al., 2019).

In contrast, endogenous activation of astrocyte CB1Rs leads to synaptic potentiation. The retrograde diffusion of endogenous cannabinoids following postsynaptic depolarization-induced production causes inhibition of subsequent neurotransmitter release from presynaptic terminals that express CB1Rs, a phenomenon known as depolarization-induced suppression of inhibition (DSI) or excitation (DSE) (Maejima et al., 2001; Wilson and Nicoll, 2001; Alger, 2002; Kreitzer et al., 2002; Ohno-Shosaku et al., 2002). An investigation for a role of hippocampal astrocytes in endocannabinoid-mediated short-term plasticity found that while a 5 s depolarization of CA1 pyramidal neurons to 0 V induces homoneuronal DSE of Schaffer collateral input, neighboring pyramidal neurons experience heteroneuronal synaptic potentiation (Navarrete and Araque, 2010). Several lines of evidence pointed at astrocyte CB1Rs being responsible for the tens of seconds lasting synaptic potentiation. This was abolished following depletion of intracellular Ca^2+^ stores with thapsigargin, while DSE was not. Also, it was inhibited by loading astrocytes with the high-affinity Ca^2+^ chelator BAPTA or the G-protein inhibitor GDPβS, a procedure leading toward enhanced DSE. While pyramidal neuron depolarization-induced astrocyte Ca^2+^ elevations were insensitive to inhibition of mGlu_1_, synaptic potentiation was inhibited, suggesting presynaptic mGlu_1_ activation downstream of astrocyte Ca^2+^ elevation is responsible for synaptic potentiation (Navarrete and Araque, 2010).

Recently, it has been demonstrated that novel object recognition is strongly impaired in astroglia-specific CB1R knockout mice (Robin et al., 2018). Hippocampal NMDA receptor function was found to be required for novel object recognition, and both NMDA receptors as well as astrocyte CB1Rs were required for *in vivo* high-frequency stimulation-induced long-term potentiation (LTP). Consistent with the dependence on NMDA receptors, high-frequency stimulation-induced LTP as well as novel object recognition could be rescued in astroglia-specific CB1R knockout mice by substituting with saturating concentration of D-serine (Robin et al., 2018). This finding suggests that astrocyte CB1R signaling is important for hippocampal LTP and novel object recognition via controlling the availability of extracellular D-serine. This conclusion, however, may have some interesting implications. As discussed earlier, it has recently been found that circadian/wakefulness-dependent cholinergic signaling through hippocampal astrocytes controls mean extracellular availability of D-serine (Papouin et al., 2017). Six hours into the active phase in environmental enrichment housing, extracellular D-serine levels saturate the glycine binding site of NMDA receptors, whereas the occupancy of this site reaches a minimum toward the end of the sleep phase (Papouin et al., 2017). The novel object recognition study here was conducted 7–10 h into the sleep phase, and indeed NMDA receptors were not saturated with endogenous D-serine (Robin et al., 2018). Therefore, it will also be important to explore the impact of hippocampal astrocyte CB1R signaling on novel object recognition during the active phase when animals are more likely to encounter object recognition challenges.

### GABAergic Signaling

In the hippocampal CA3-CA1 microcircuit, astrocyte GABA_*B*_ receptor signaling plays a role in counterbalancing GABAergic interneuron-mediated inhibition of Shaffer collateral glutamate release (Perea et al., 2016). At states of low interneuron excitation (individual action potential firing) Shaffer collateral glutamate release is inhibited in a GABA_*A*_ receptor-dependent manner. In contrast, when interneurons fire bursts of action potentials, GABA_*B*_ receptors on astrocytes are recruited, as astrocyte-specific knockout experiments confirmed, leading to enhanced Ca^2+^ signaling and group 1 mGlu receptor-dependent enhancement of Shaffer collateral glutamate release. Astrocyte-specific GABA_*B*_ receptor knockout was sufficient to reduce the power of hippocampal theta and low gamma oscillations (30–60 Hz) in anesthetized mice (Perea et al., 2016). The contribution of astrocyte-dependent Ca^2+^ signaling to low gamma oscillations is consistent with findings from transgenic mice with astrocyte-selective overexpression of tetanus toxin (Lee et al., 2014). Here the reduction of low gamma oscillations was, however, only significant in awake mice, not during sleep.

A recent study explored the circuit and behavioral consequences of astrocyte-specific knockout of GABA_*B*_ receptors in prefrontal cortex (Mederos et al., 2021). They also found a reduction of low gamma oscillations in active awake mice, but not in resting mice. Low gamma oscillations may reflect synchronization of the neuronal population and has been associated with working memory processing in prefrontal cortex (Buzśaki and Wang, 2012). Indeed, mice lacking GABA_*B*_ receptors in prefrontal cortex astrocytes have difficulty making correct decisions during the T-maze alternating behavioral task, a paradigm testing working memory performance (Mederos et al., 2021). A more detailed cellular analysis of the circuit function revealed that astrocyte GABA_*B*_ receptors, IP_3_R2-dependently, potentiate parvalbumin-positive interneuron-driven inhibitory postsynaptic currents in local neurons. Using astrocyte-specific overexpression of melanopsin^1^ for optical control of G_*q*_ protein-coupled receptor activation exclusively in astrocytes (Mederos et al., 2019), they not only rescued goal-directed behavior in astrocyte-GABA_*B*_ receptor knockout mice but further enhanced goal-directed behavior in wildtype mice (Mederos et al., 2021). This observation is in contrast with disruptive consequences of astrocyte-specific activation of exogenous Gi protein-coupled receptors in striatal astrocytes, which also enhances Ca^2+^ dynamics but leads to hyperactivity of the mice (Nagai et al., 2019). It indicates that brain region, subcellular location of receptors within astroglia, and duration of activation likely all have inherent importance.

## Intrinsic Microdomain Signaling

The mitochondrial PTP appears to be a major determinant of spontaneous, intrinsically regulated astroglia microdomain Ca^2+^ fluctuations (Agarwal et al., 2017). The intrinsic nature of these signaling events likely stems from metabolic control (Jackson and Robinson, 2015; Agarwal et al., 2017; Oheim et al., 2018). In addition, the promiscuous regulation of mPTP activity by ROS, Ca^2+^, and IP3R among others suggests that stochastic activation of the many G protein-coupled astrocyte receptors in the presence of ambient receptor ligands at low basal concentrations or in their absence may account for the considerable bafilomycin A1-insensitive, IP_3_R2-dependent portion of microdomain Ca^2+^ events (Agarwal et al., 2017). To further this complexity, vigilance/arousal-dependent, predominantly noradrenergic signaling, which is critical for whole-cell, widespread Ca^2+^ elevations (Ding et al., 2013; Paukert et al., 2014; Ye et al., 2020), also contributes to microdomain events (Agarwal et al., 2017), as does local neuronal activity through activation of various receptors or ion channels (Agarwal et al., 2017; Bindocci et al., 2017; Stobart et al., 2018). The complexity of astroglia microdomain Ca^2+^ dynamics so far has made it impossible to genetically manipulate selectively microdomain activity while leaving basal Ca^2+^ as well as whole-cell Ca^2+^ elevations intact. More refined mechanistic insight into what happens in astroglia mitochondria during distinct behavioral and environmental states will be required to design gentle but highly specific genetic manipulations.

In the midst of this challenge, the *Drosophila* model system offers an opportunity to selectively target astrocyte microdomain Ca^2+^ events (Ma and Freeman, 2020). In contrast to mammalian microdomain events, apart from the ineffective neurotransmitters including glutamate, acetylcholine, GABA and octopamine, only tyramine, which corresponds to mammalian dopamine, enhances the frequency of *Drosophila* TrpML-mediated Ca^2+^ transients by approximately 40%. Around half of the microdomain events are associated with the tracheal system, which in *Drosophila*, consists of a network that terminates in filopodia throughout the nervous system and facilitates oxygen delivery. Retractions of tracheal filopodia are often preceded by astrocyte microdomain events. Inhibiting or genetically deleting TrpML in astrocytes eliminates microdomain Ca^2+^ events and increases ROS concentration and results in growth of the tracheal system, suggestive of reduced filopodia retractions (Ma and Freeman, 2020). Together, these findings suggest that astroglia microdomain Ca^2+^ dynamics are not only an evolutionary conserved phenomenon, but may serve a conserved functional role as homeostatic element within the metabolic state of the microenvironment. As we will discuss below, a way of studying preferentially microdomain Ca^2+^ events in mammalian astroglia could be represented by sleep studies, where vigilance/arousal-dependent whole cell Ca^2+^ events, like under general anesthesia, are strongly reduced, if not absent (Bojarskaite et al., 2020; Ingiosi et al., 2020).

## Implications of Astroglia Basal Ca^2+^ for Local Circuit Excitability

*Drosophila* astroglia have granted insight into diverse mechanisms of how basal Ca^2+^ controls local circuit excitability and behavior. In cortex glia, which are located in brain areas densely populated with cell bodies but no synapses, the genetic deletion of the Na^+^/Ca^2+^, K^+^ exchanger *zydeco* leads to elevation of intracellular Ca^2+^ accompanied by a proneness to seizure activity (Melom and Littleton, 2013). The constant elevation of Ca^2+^ causes the intrinsic microdomain Ca^2+^ activity to disappear, possibly through masking it. Intracellular Ca^2+^ elevation in cortex glia is mechanistically linked to seizure activity by activation of calcineurin, subsequent endocytosis of two-pore K^+^ channel *sandman*, and impaired extracellular buffering of K^+^ (Weiss et al., 2019). The elevated extracellular K^+^ will depolarize surrounding excitable cells and bring their membrane potential closer to the threshold of excitation.

In *Drosophila* astrocytes, which are located in areas containing synapses, intracellular Ca^2+^ elevation triggers a different signaling pathway resulting in paralysis (Zhang et al., 2017). It was found that Ca^2+^ elevation increases the expression of Rab11, a GABA plasma membrane transporter (GAT) regulator, facilitating endocytosis of GAT, impairing synaptic GABA clearance, and leading to paralysis (Zhang et al., 2017). An analogous mechanism was uncovered in mouse striatal astrocytes where experimental reduction of intracellular Ca^2+^ leads to enhanced GABA clearance and reduced tonic inhibition of the surrounding microcircuitry, ultimately causing excessive self-grooming (Yu et al., 2018). Mouse astroglia can also control tonic inhibition through GABA release via Ca^2+^-activated anion channel bestrophin-1. This mechanism has been proposed for cerebellar Bergmann glia controlling excitability of granule cells (Lee et al., 2010) and for astrocytes in the striatum controlling excitability of medium spiny neurons (Yoon et al., 2014). Astroglia-mediated tonic inhibition has been associated with limited performance on the rotarod paradigm (Woo et al., 2018). Also, bestrophin-1 has been implicated in vesicle-independent astrocytic release of glutamate and D-serine (Oh and Lee, 2017).

## Consequences of Deletion of IP_3_R2 Signaling

IP_3_R2 is an astroglia Ca^2+^ signaling junction point. It is by far the most abundant IP_3_R subtype in astroglia (Sharp et al., 1999; Holtzclaw et al., 2002; Petravicz et al., 2008). Upon gene deletion, whole-cell Ca^2+^ elevations are almost completely abolished and also the frequency of microdomain events is considerably reduced (Fiacco et al., 2007; Srinivasan et al., 2015; Agarwal et al., 2017), suggesting that through its interaction with mPTP, it is a major pathway how G protein-coupled receptors facilitate microdomain events. The importance of IP_3_R2 for astroglia Ca^2+^ dynamics is further emphasized by the finding that it serves not only G_*q*_ protein-coupled receptors but also G_*i/o*_ protein-coupled receptors for intracellular Ca^2+^ release (Durkee et al., 2019). Given the apparent importance of IP_3_R2 for astroglia Ca^2+^ dynamics, it came as a surprise that a series of acute hippocampal slice studies of baseline and stimulated synaptic function and synaptic plasticity found no consequences of IP_3_R2 gene deletion (Fiacco et al., 2007; Petravicz et al., 2008; Agulhon et al., 2010). These findings triggered more refined slice physiology studies that applied astrocyte-specific knockout of endogenous receptors and the dissection of concomitant excitatory as well as inhibitory or potentiating as well as depressing circuit mechanisms (e.g., Perea et al., 2016). In addition, the study of spontaneous as well as agonist-induced hippocampal astrocyte Ca^2+^ dynamics from acute slices of mice carrying a combined gene deletion of IP_3_R2 and IP_3_R3 suggests that non-type 2 IP_3_Rs may contribute to astroglia Ca^2+^ elevations (Sherwood et al., 2017). Not only at the level of slice physiology, also at the behavioral level the consequences of IP_3_R2 gene deletion have been less impressive than one might have expected. A battery of behavioral tests with astrocyte-specific IP_3_R2 gene deletion mice revealed no significant difference to wildtype littermates when testing anxiety, depressive behavior, motor coordination, exploratory behavior, sensory motor gating with the acoustic startle response and prepulse inhibition, and with learning and memory using the Morris water maze (Petravicz et al., 2014).

On the other hand, recent years have also seen a considerable number of findings demonstrating how loss of IP_3_R2 function does affect developmental synaptic pruning, neurovascular coupling, cortical plasticity and memory, and the regulation of sleep. Similar to the climbing fiber synapse in the cerebellum mentioned earlier (Iino et al., 2001), the principal trigeminal nucleus-ventral posteromedial thalamic nucleus synapse serves as a model of developmental synaptic pruning since between postnatal day 7 (P7) and P16, 7–8 inputs are refined into a single mature input (Yang et al., 2016). Using a combination of acute slice electrophysiology and immunocytochemistry it has been found that this synapse elimination is incomplete in *ITPR2^–/–^* mice, leaving on average more than two functional inputs at P16. Local administration of ATP or the P2Y_1_ receptor agonist MRS-2365 rescued synapse elimination, but global P2Y_1_ receptor gene deletion made all rescue attempts fail (Yang et al., 2016). This suggests a model where IP_3_R2-dependent astrocytic ATP release acts through P2Y_1_ receptors to facilitate synapse elimination.

In awake behaving mice, learning and memory can be controlled by astroglia Ca^2+^ dynamics at different time scales. Padmashri et al. (2015) used an inducible astroglia-specific IP_3_R2 gene deletion approach to test whether motor skill learning is affected. Even though the conditional knockout approach reduced astrocyte Ca^2+^ responses to the application of ATP only by approximately 50%, knockout mice reached an earlier ceiling of the success rate in a forelimb reaching-task after 3 of 5 days. They also found a linear relationship between residual Ca^2+^ responsiveness and preservation in motor skill learning (Padmashri et al., 2015). Pinto-Duarte et al. (2019) did not find an effect of global IP_3_R2 gene deletion on novel object recognition, fear memory, or spatial memory within 2–5 days. But in all three tests, they found a considerable impairment 2–4 weeks later. Since such a remote memory deficit might represent impaired memory consolidation, they investigated hippocampal Schaffer collateral-to-CA1 field potential LTD in acute slices. They found impaired LTD in *ITPR2^–/–^* mice, which could be rescued with exogenous D-serine, but not when preincubated with the degrading enzyme D-amino acid oxidase (DAAO) or with NMDA receptor inhibition (Pinto-Duarte et al., 2019). Astroglia Ca^2+^ dynamics can also acutely shape cortical neuronal population activity in response to sensory stimulation (Lines et al., 2020). Monitoring somatosensory cortex gamma activity revealed that sensory stimulation induces a transient increase in power that attenuates to a steady-state level for the duration of the stimulation. In *ITPR2^–/–^* mice, the attenuation is diminished. This is unlikely due to developmental changes, since acute elevation of astroglia Ca^2+^ via astroglia-specific activation of hM3Dq reduces the gamma activity (Lines et al., 2020).

Several studies have focused on the role of astroglia Ca^2+^ signaling during sleep. A very elegant recent study combined monitoring of somatosensory cortex astrocyte Ca^2+^ dynamics, locomotion activity of head-fixed mice, whisking activity, as well as cortical electrical activity and muscle activity in *ITPR2^–/–^* mice and wildtype littermates (Bojarskaite et al., 2020). In the analysis of sleep states, they distinguished between slow wave sleep with a further distinction between non-rapid eye movement (NREM) sleep and intermediate state (IS) sleep, and rapid eye movement (REM) sleep. As expected for a behavioral state lacking significant arousal, astrocyte Ca^2+^ dynamics during sleep are dominated by microdomain events. Preceding the state transition from either of the two slow wave sleep states (NREM or IS) to wakefulness, there is an enhanced IP_3_R2-dependent Ca^2+^ elevation. This finding is consistent with an independent recent astroglia Ca^2+^ imaging study where intracellular Ca^2+^ release was attenuated by astroglia-specific gene deletion of stromal interaction molecule 1 (STIM1) (Ingiosi et al., 2020). The transition from slow wave sleep to wakefulness is when enhanced norepinephrine release is expected (Takahashi et al., 2010). Surprisingly, neither study found a Ca^2+^ elevation during REM sleep (Bojarskaite et al., 2020; Ingiosi et al., 2020) when elevated levels of extracellular acetylcholine are expected (Jasper and Tessier, 1971). This finding, together with the inhibition of global widespread cortical astroglia Ca^2+^ elevations during awake states of heightened vigilance by α_1_-adrenergic receptor antagonists (Ding et al., 2013; Srinivasan et al., 2015) raise the possibility that acetylcholine cannot directly cause significant cortical astroglia Ca^2+^ elevations. Another finding in *ITPR2^–/–^* mice was increased frequency of sleep spindles during IS sleep, which may cause impaired memory consolidation (Bojarskaite et al., 2020). This finding may provide a mechanistic explanation for impaired remote memory in *ITPR2^–/–^* mice discussed above (Pinto-Duarte et al., 2019) or for impaired remote memory in consequence of manipulation of astroglia Ca^2+^ dynamics using the humanized G_*i*_ protein-coupled muscarinic receptor type 4 DREADD (hM4Di) discussed below (Kol et al., 2020). Bojarskaite et al. (2020) also found considerably reduced delta electrocorticogram power during NREM sleep in *ITPR2^–/–^* mice, which indicates reduced sleep pressure, which is consistent with the first study that reported astrocyte control of sleep pressure and the impact of sleep deprivation (Halassa et al., 2009). This study by Halassa et al. (2009) used astrocyte-specific overexpression of dn-SNARE to impair the proposed vesicular release of ATP and to reduce occupancy of adenosine A1 receptors, which are important for mediating sleep pressure. Consistent findings on sleep pressure and sleep deprivation were obtained by Ingiosi et al. (2020). Finally, Bojarskaite et al. (2020) found that loss of IP_3_R2 function reduces the dwell time but increases the frequency of both slow wave sleep states, leaving REM sleep unaltered. This finding is almost opposite to the outcome of a study where IP3 signaling was directly attenuated by astroglia-specific overexpression of the venus-tagged IP3 5’phosphatase (VIPP) (Foley et al., 2017). There they found an increased number of REM sleep bouts with no difference in dwell time and no change in NREM sleep. The discrepancy in results may be explained by the fact that IP_3_R2 gene deletion will considerably attenuate intracellular Ca^2+^ release mediated by possibly all astrocyte G protein-coupled receptors. In contrast, hydrolysis of IP3 is not expected to interfere with Gi/o βγ subunit binding to IP3R2. Therefore, it is tempting to speculate that activation of α_2_-adrenergic receptors, or of any other astrocyte Gi/o protein-coupled receptor in the study by Foley et al. (2017) could account for the difference in sleep regulation among these two studies.

## Consequences of Astroglia-Specific Manipulation of Ca^2+^ Dynamics Via Exogenous Actuators

In recent years, astroglia-specific overexpression of exogenous actuators for manipulation and interrogation of astroglia physiology has become very popular (Table 2). The advantages of such approaches are immediately clear since almost all receptors on astroglia are also present on neurons and other neural cells. Limitations of the utility of exogenous actuators need to be considered in light of the downstream signaling events that follow the astroglia-specific initiation. Actuators which remove an endogenous signal may arguably be regarded as less invasive. One example is the CalEx constitutively active plasmalemmal Ca^2+^ pump and will be discussed below. For actuators that represent the overexpression of an exogenous G protein-coupled receptor that elevates Ca^2+^, one needs to consider the physiological dynamic range of the expected signal regarding expression level, its subcellular location, and duration/pattern of ligand application. Predictions of the consequences of overexpression of ion channels, some with ion selectivity for which no physiological counterpart is known in astroglia, a cell type with ion buffering function, are most complex. Nevertheless, these are very powerful tools that may inform follow-up studies focused on the physiological role of specific endogenous astroglia signaling pathways.

To inhibit microdomain events despite their mechanistic diversity, Yu et al. (2018) modified human plasma membrane Ca^2+^ ATPase pump (hPMCA) isoform 2, w/b splice variant with excised cytosolic interaction domains (hPMCA2w/b), resulting in a modified pump that constitutively extrudes cytosolic Ca^2+^. The model was coined “CalEx” due to its function in Ca^2+^ extrusion^2^ (Yu et al., 2018). Behavioral assays of mice expressing CalEx in striatal astrocytes revealed that reducing whole-cell and microdomain Ca^2+^ dynamics results in excessive self-grooming behavior indicative of an obsessive-compulsive disorder phenotype. The underlying circuit mechanism was identified as low astrocyte Ca^2+^ leading to reduced expression of Rab11a, which leads to less internalization of the GABA transporter GAT-3, more GABA uptake, lower ambient GABA concentration and less tonic inhibition (Yu et al., 2018). This mechanism is conserved in *Drosophila* astrocytes (Zhang et al., 2017), but the relationship between basal mouse astrocyte Ca^2+^ set by TRPA1 and surface expression of GAT-3 in co-culture with neurons is reversed (Shigetomi et al., 2011). CalEx overexpressed in mouse prefrontal cortex astrocytes was used in comparison with hM3Dq^3^, the G_*q*_ protein-coupled DREADD, to study the effects of manipulating astrocyte Ca^2+^ on ethanol’s motivational, stimulatory and sedating impact (Erickson et al., 2020). It was found that while boosting astrocyte Ca^2+^ dynamics enhances all negative effects such as ethanol consumption, excessive locomotor stimulation in response to low dose ethanol and prolonged loss of righting reflex (LORR) due to high dose ethanol sedation, reducing astrocyte Ca^2+^ dynamics with CalEx has protective, opposite effects. The aggravated sedating effect of enhanced astrocyte Ca^2+^ could be reversed by inhibiting adenosine A1 receptors (Erickson et al., 2020), suggesting that hM3Dq activation in prefrontal cortex astrocytes may trigger ATP release. In the light of these findings it is interesting to note that the suppression of vigilance-dependent astroglia Ca^2+^ activation by ethanol inhibition of norepinephrine release (Ye et al., 2020) may play a homeostatic protective role during acute ethanol intoxication.

The Goshen group recently expressed hM4Di^4^ in hippocampal CA1 astrocytes to inhibit their Ca^2+^ elevations (Kol et al., 2020). This is remarkable, because Gi/o protein-coupled receptors usually enhance Ca^2+^ dynamics in astroglia. Others indeed found that hM4Di when expressed in hippocampal CA1 astrocytes enhances Ca^2+^ signals (Durkee et al., 2019), and this is consistent with striatal astrocytes (Nagai et al., 2019). One could imagine two possible explanations for this discrepancy. It is possible that the expression level, which could then also affect the subcellular location, was different. Or, since they used the mode in order to present the quantification of Ca^2+^ events, which represents the global maximum in the amplitude frequency distribution but completely ignores local maxima, it may be interesting to see the full distribution in response sizes. Nevertheless, in this elegant study they found that the CA1-to-anterior cingulate cortex (ACC) projection is critical for remote memory but not for recent memory. Inhibiting CA1 astrocyte Ca^2+^ dynamics then disrupts remote memory (Kol et al., 2020). These results are consistent with the recent finding that IP_3_R2 gene deletion impairs remote but not recent memory (Pinto-Duarte et al., 2019).

The mas-related genes G_*q*_ protein-coupled orphan receptor MrgA1, which is naturally only expressed in some nociceptive neurons (Han et al., 2002), was the first xenoreceptor used for astroglia-specific Ca^2+^ activation (Fiacco et al., 2007). When expressed in cerebellar Bergmann glia of anesthetized mice, it was found that application of its peptide ligand, the Phe-Met-Arg-Phe (FMRF)amide, a whole-cell Ca^2+^ elevation is accompanied by extracellular reduction in potassium concentration leading to a temporary hyperexcitability of Purkinje cells, the cerebellar principal neurons (Wang et al., 2012); however, see Smith et al. (2018). A different study used the hyperpolarizing optogenetic actuator Archaerhodopsin (Arch)^5^ in cortical astrocytes of awake mice and found that Arch light-activation causes Ca^2+^ elevations that, in terms of their spatial restriction, resemble microdomain events, but last tens of seconds (Poskanzer and Yuste, 2016). The mechanism of how Arch leads to Ca^2+^ elevation remains elusive. As a consequence of astrocyte Arch activation, extracellular glutamate spikes can be detected accompanying slow oscillatory and more synchronized neuronal activity (Poskanzer and Yuste, 2016).

Activation of hM3Dq and the α_1_-adrenergic receptor-based opsin Opto-Gq (Airan et al., 2009) in hippocampal CA1 astrocytes to elevate Ca^2+^ has been demonstrated to be sufficient to induce NMDA receptor-dependent LTP and contextual memory (Adamsky et al., 2018). In contrast, hM3Dq activation in neurons has a detrimental effect on memory performance (Adamsky et al., 2018). Activation of hM4Di in striatal astrocytes leads to enhanced Ca^2+^ elevations and hyperactivity (Nagai et al., 2019). Transcriptomic analysis of activated astrocytes revealed the reactivation of thrombospondin 1, which is an important factor during developmental synaptogenesis (Christopherson et al., 2005). Treatment with gabapentin, antagonist of the thrombospondin 1 receptor, rescues all morphological as well as all functional changes (Nagai et al., 2019). They further demonstrated that hM4Di activation in astrocytes can be used to compensate phenotypically for mouse disease models which display reduced astrocytic Ca^2+^ activity, such as the Huntington’s disease mouse model (Yu et al., 2020). Durkee et al. (2019) conducted a systematic comparison of the effects of hM3Dq and hM4Di when expressed in neurons or astrocytes. They found that hM4Di, like GABA_*B*_R activation, elevates Ca^2+^ levels in astrocytes in an IP_3_R2-dependent manner. Yet, the phospholipase C inhibitor U73122 failed to inhibit hM4Di as well as GABA_*B*_R responses in astrocytes. This suggests the possibility that in general, Gi/o protein-coupled receptors in astroglia couple via the βγ subunit to the IP_3_R2 (Zeng et al., 2003; Durkee et al., 2019).

## Outlook

**FIGURE 1:**
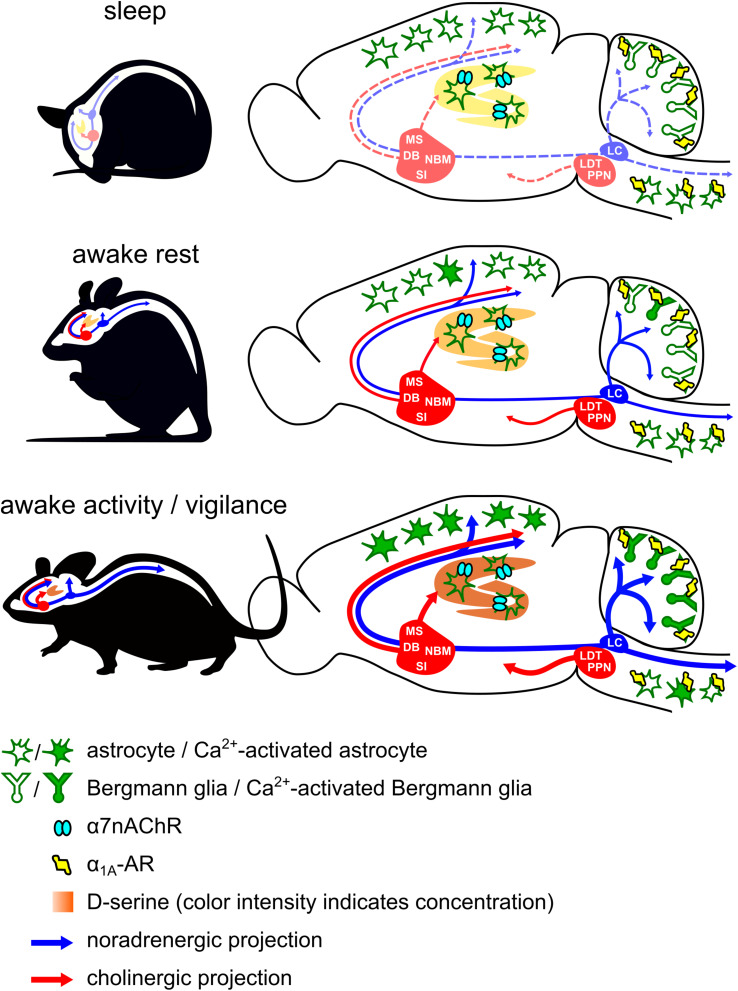
Diagram of behavioral state-dependent activation of noradrenergic and cholinergic systems and their modulation of astroglia Ca^2+^ dynamics during sleep, rest, and vigilance. Astrocytes in primary visual cortex and somatosensory cortex, *Hes5*-positive astrocytes in spinal dorsal horn, and cerebellar Bergmann glia exhibit norepinephrine-dependent global Ca^2+^ rises during states of heightened vigilance. In Bergmann glia and *Hes5*-positive astrocytes of spinal dorsal horn, this arousal-associated Ca^2+^ signal is mediated by α_1*A*_-adrenergic receptors. Cholinergic activation of hippocampal astrocytes through α7 nicotinic acetylcholine receptors has been found to modulate D-serine concentration depending on the sleep-wake state. LC, locus coeruleus; LDT, laterodorsal tegmental nucleus; PPN, pedunculopontine nucleus; MS, medial septal nucleus; DB, diagonal band of Broca; NBM, nucleus basalis of Meynert; SI, substantia innominata.

Considerable progress has been made in our understanding of the role of astroglia Ca^2+^ dynamics for the processing of information in the brain. Behavioral state transitions occur constantly, ranging from different sleep states to various levels of vigilance during wakefulness and are disrupted in psychiatric disease. Emerging experimental findings, which are schematized in Figure 1, emphasize the importance of the particular behavioral state that determines which molecular mechanisms predominate the respective astroglia Ca^2+^ signal, what spatial and temporal pattern it assumes, and how it feeds back to behavior. While the precise astroglia Ca^2+^ signals in unanesthetized animals have not yet been defined *in vivo* for many of the discussed receptors, it appears that a significant portion of astroglia Ca^2+^ dynamics are representations of neuromodulator signaling and are primed for a role in informing neural populations about the behavioral and environmental context. Since dependence of astroglia Ca^2+^ activation on behavioral state relies on variable release of neuromodulator and not on variable intrinsic responsiveness of astroglia, it is possible that exogenous astroglia-specific actuators have led to more robust phenotypes because the limited spatial and temporal precision of this approach allows the recruitment of astroglia Ca^2+^ activation out of behavioral or environmental context. On the other hand, it is encouraging that several similarities in phenotypes have been independently defined. For example, the importance of astroglia Ca^2+^ dynamics in memory consolidation allowing remote memory formation has been discovered using the IP_3_R2 gene deletion model as well as independently using inhibitory DREADD overexpression in hippocampal astrocytes. Similarly, a role for astroglia Ca^2+^ dynamics in developmental synapse elimination was discovered using the same IP_3_R2 gene deletion model, and independently it was found that developmental synaptogenic cues can be reactivated by exogenous enhancement of astroglia Ca^2+^ dynamics in adult mice. It will be important to combine insight from exogenous activation studies with our increasing understanding of the behavioral and environmental context when endogenous astroglia signaling pathways are naturally engaged to revise and customize conventional behavioral paradigms for testing the physiological impact of astroglia Ca^2+^ dynamics. This will be a prerequisite for recognizing alterations in astroglia Ca^2+^ dynamics and for understanding how they can contribute to nervous system disease.

## Author Contributions

All authors listed have made a substantial, direct and intellectual contribution to the work, and approved it for publication.

## Conflict of Interest

The authors declare that the research was conducted in the absence of any commercial or financial relationships that could be construed as a potential conflict of interest.

## Footnotes

1 https://www.addgene.org/122630/

2 http://www.addgene.org/111568/

3 https://www.addgene.org/50478/

4 https://www.addgene.org/50479/

5 https://www.addgene.org/28307/
